# TG: HDL-C Ratio as Insulin Resistance Marker for Metabolic Syndrome in Children With Obesity

**DOI:** 10.3389/fendo.2022.852290

**Published:** 2022-03-10

**Authors:** Ahmad Kamil Nur Zati Iwani, Muhammad Yazid Jalaludin, Abqariyah Yahya, Fazliana Mansor, Fuziah Md Zain, Janet Yeow Hua Hong, Ruziana Mona Wan Mohd Zin, Abdul Halim Mokhtar

**Affiliations:** ^1^ Endocrine and Metabolic Unit, Institute for Medical Research, Ministry of Health Malaysia, Setia Alam, Malaysia; ^2^ Department of Pediatrics, Faculty of Medicine, University Malaya, Kuala Lumpur, Malaysia; ^3^ Department of Social and Preventive Medicine, Faculty of Medicine, University Malaya, Kuala Lumpur, Malaysia; ^4^ Department of Pediatrics, Hospital Putrajaya, Ministry of Health Malaysia, Putrajaya, Malaysia; ^5^ Department of Sports Medicine, Faculty of Medicine, University Malaya, Kuala Lumpur, Malaysia

**Keywords:** pediatric, obesity, TG: HDL-C ratio, metabolic syndrome, insulin resistance

## Abstract

Insulin resistance (IR) is an important variable in the diagnosis of metabolic syndrome (MetS). Currently, IR is not part of the existing pediatric definition of MetS, instead elevated fasting blood glucose (FBG) is measured as an indicator of hyperglycemia. Arguably, many obese children with severe IR are still able to regulate their FBG well. Hence, this study aimed to assess the utility of triglyceride-to-high-density lipoprotein cholesterol (TG : HDL-C) ratio as an IR marker in the modeling of pediatric MetS among children with obesity using structural equation modeling (SEM). A total of 524 blood samples from children with obesity (age 10–16 years old) were analyzed for FBG, lipids, insulin, leptin, and adiponectin. Both exploratory (EFA) and confirmatory factor analysis (CFA) were used to examine TG : HDL-C ratio as an IR marker in pediatric MetS. EFA shows that TG: HDL-C ratio (standardized factor loading = 0.904) groups together with homeostasis model assessment-estimated insulin resistance (HOMA-IR) (standardized factor loading = 0.664), indicating a strong correlation to the IR factor. Replacing FBG with TG: HDL-C ratio improved the modeling of MetS structure in children with obesity. Our MetS model of TG: HDL-C ratio as IR component shows comparable model fitness indices (goodness of fit, Akaike’s information criterion, and Bayesian information criterion) with leptin:adiponectin ratio (platinum standard for adiposity:IR marker) model. The least model fit was seen when using FBG as an IR surrogate. TG : HDL-C ratio performed better as IR surrogate in MetS structures (standardized factor loading = 0.39) compared to FBG (standardized factor loading = 0.27). TG: HDL-C ratio may be considered as an IR component in pediatric MetS.

## 1 Introduction

Metabolic syndrome (MetS) is a cluster of risk factors that includes obesity, dyslipidemia, insulin resistance (IR) or impaired glucose tolerance, and elevated blood pressure (BP). The significance of MetS among pediatrics arises in line with the growth of obesity prevalence among children and the rise of MetS in adults. Early identification and treatment of obese children and adolescents with multiple metabolic derangements, particularly those at higher risk, may curb the risk of developing cardiometabolic diseases such as cardiovascular disease (CVD) and type 2 diabetes mellitus (T2DM). However, identifying those who are affected is rather difficult because clear recommendations about how to diagnose MetS in the young age group are still lacking (1). Among others, one of the limitations is the reliance on elevated fasting blood glucose (FBG) rather than fasting insulin or the homeostasis model assessment-estimated insulin resistance (HOMA-IR) as a measure of impaired glucose regulation whereby many children with severe IR can still regulate their FBG (2). Therefore, there is a need to search for a simple, reliable, and applicable surrogate marker to measure IR among children to improve the existing pediatric MetS definition.

A large multi-ethnic cohort study by Giannini et al. (3) has proposed the use of triglyceride-to-high-density lipoprotein cholesterol (TG: HDL-C) ratio as a cheap and reliable IR surrogate in children with obesity. This study (3) showed some limitations in evaluating the TG : HDL-C ratio as an IR marker, and it was specific to the US population. South Asians are more IR than Caucasians and African-Americans (4), and therefore, TG : HDL-C ratio has more clinical potential in the diagnosis of MetS in this population. Although previous studies have demonstrated the use of TG : HDL-C ratio as an IR marker and identifying children at risk for MetS in the West (5, 6) and Asia (7, 8), none of the studies employed structural equation modeling (SEM) to validate and measure the strength of correlation between TG: HDL-C ratio and IR in the theoretical structure of MetS.

Pediatric MetS diagnosis was based on the grouping of intercorrelated factors and variables that will introduce multicollinearity, which violates one of the conventional regression model assumptions. Factor analysis is a distinctive feature of SEM in which a series of dependence relationships can be examined simultaneously in one technique accounting for measurement error. This type of statistical analysis is not possible in multiple regression analysis. In a typical multiple regression analysis, the association was measured between a single dependent variable and multiple covariates. These covariates are assumed to be measured without measurement error (9). Factor analysis has been used in numerous MetS studies (10, 11) particularly in establishing the parameters used to measure each MetS risk factor such as the use of body mass index (BMI), waist-to-hip ratio, and waist circumference (WC) as measures of obesity (12, 13). Furthermore, the recently emerging MetS scoring was established using factor analysis (14, 15).

To our knowledge, none of the previous pediatric MetS studies has employed SEM in determining the strength of correlation between TG : HDL-C ratio and IR in the theoretical structure of MetS. We hypothesized that TG: HDL-C ratio is highly correlated with measures of IR (insulin and HOMA-IR) and will group together as IR group in the MetS structure. Secondly, we also hypothesized that TG : HDL-C ratio is a better IR surrogate in the MetS structure than FBG. Thirdly, given that children’s obesity is homogeneous in this study, the hypothesized model was referenced to a model of an established adiposity-IR marker (leptin:adiponectin ratio). Therefore, the main objective of this study is to examine the utility of TG: HDL-C ratio as an IR marker in the MetS structure of children with obesity.

## 2 Materials and Methods

### 2.1 Study Design and Participants

**Figure 1:**
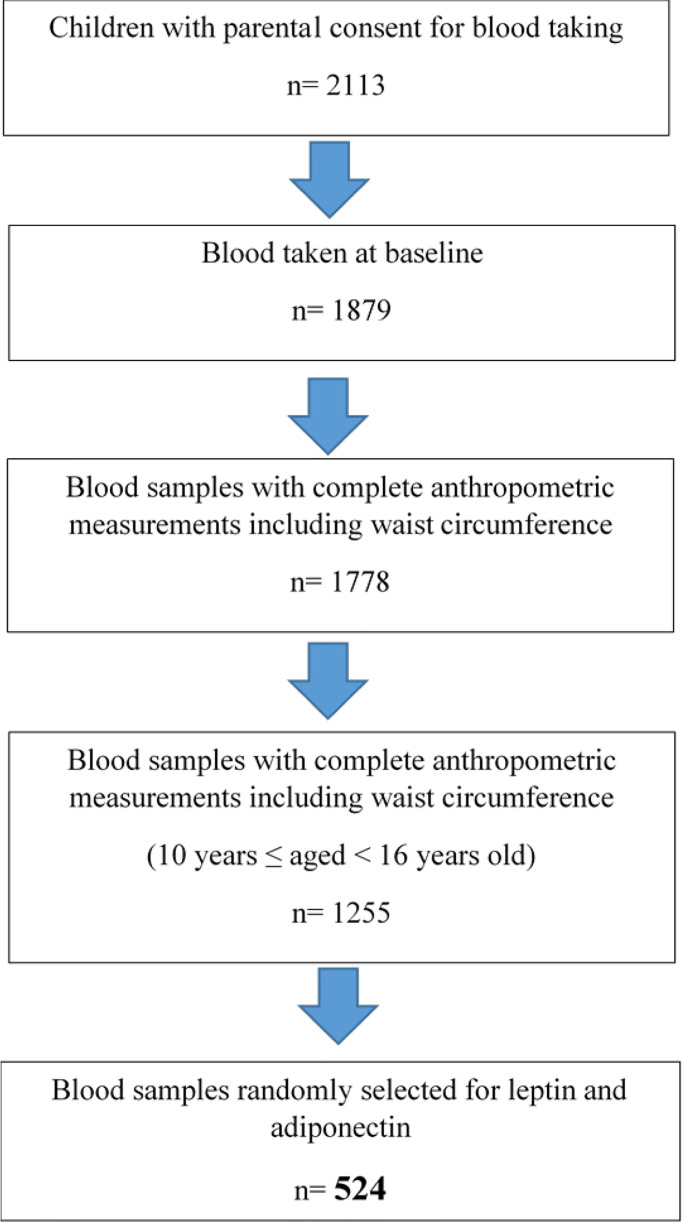
Flowchart of blood selection for leptin and adiponectin testing.

This study was performed using a cross-sectional baseline data of children with obesity participating in the My Body is Fit and Fabulous at School (MyBFF@school) programme, a school-based cluster randomized controlled trial (C-RCT) study. Detailed descriptions of the recruitment of MyBFF@school programme have been previously published (16). In general, MyBFF@school was designed to address the rise of childhood obesity among Malaysian schoolchildren. MyBFF@school was conducted for 6 months between February 2016 and August 2016 in the Federal Territory of Kuala Lumpur, Selangor, and Negeri Sembilan of Malaysia. For this study, we randomly selected 524 baseline blood samples from children with obesity older than 10 years but below 16 years old with complete anthropometric measurements, BP measurements, and pubertal staging data (**Figure 1**). Ethical approval was granted by the Medical Research and Ethics Committee (MREC), Ministry of Health Malaysia (NMRR-18-2749-41841).

### 2.2 Health and Physical Examination

Prior to health and physical examination, children were asked to fast overnight for at least 8 h. Anthropometric measurements were performed by trained personnel, and medical officers and pediatricians performed health examinations. Standing height was measured without shoes to the nearest 0.1 cm using a calibrated stadiometer (Seca 217, Germany). Body weight and body fat mass were measured in light clothing without shoes and socks to the nearest 0.1 kg using a precalibrated body impedance analyzer (InBody 720, Korea). WC was measured twice to the nearest 0.1 cm over the skin midway between the 10th rib and the iliac crest at the end of normal expiration using a non-extensible tape (Seca 201, Germany), and the mean was recorded.

Two BP readings were measured after 5 min of rest using a mercury sphygmomanometer (Accoson, UK) seated with the arm supported at the heart level, and the mean was recorded. Pubertal status was assessed by showing a standardized Tanner staging picture to the child (17, 18). Children were also examined—by pediatricians—for the presence of acanthosis nigricans (AN) over the neck (19). AN was determined based on Burke’s quantitative dichotomous score (19).

### 2.3 Blood Sample Collection

Venipuncture was performed by trained nurses and doctors. Blood samples were collected from participating children who consented to blood taking at the participating schools whereby the processing of blood samples was kept to a minimum. Blood samples were transported cold (4°C) in a coolant box with frozen coolant to the central laboratory at the Institute for Medical Research within 2 h of collection and processed on the same day. Aliquots of serum/plasma samples were kept at -20°C and -80°C prior to analysis.

### 2.4 Biochemical Parameters

HbA1c level was determined by cationic exchanged high-performance liquid chromatography (Adams A1c HA-8160, Arkray Inc., Japan) and followed the National Glycohemoglobin Standardization Programme Guidelines. FBG, TG, total cholesterol, HDL-C, and low-density lipoprotein cholesterol (LDL-C) were analyzed using an automated analyzer (Dirui CS-400, China) with reagents purchased from Randox Laboratories (Antrim, UK).

Fasting insulin concentration was measured using an automated enzyme immunoassay analyzer (TOSOH AIA-360, Japan). Inter-assay coefficient of variability (CV) for insulin at 9.4, 53.7, and 141.8 µU/ml was 5.7%, 3.6%, and 5.2%, respectively. Serum adiponectin was measured using an automated analyzer (Dirui CS-400, China) with reagents purchased from Randox Laboratories (Antrim, UK). The inter-assay CV for adiponectin at 6.1 and 12.4 µg/ml was 9.4% and 5.6%, respectively. Serum leptin was measured by commercial ELISA assay (IBL International, Germany) in two replicates with two controls (Low and High) at each plate. The detection limit of the assay was 0.7–100 ng/ml. The inter-assay CV for low control was <10% and for high control was <15%. In general, immunoassay results are considered reliable when intra-assay CV was <10% and inter-assay CV was <15% (20).

### 2.5 Operational Definitions of Study Variables

#### 2.5.1 Obesity Status

Overweight and obesity were defined as BMI z-score above 1 and 2 standard deviations for age and sex according to the WHO BMI chart (21).

#### 2.5.2 Pubertal Staging

Stage 1 external genitalia development and breast development for boys and girls were classified as prepubertal, while stage 2 and above were defined as pubertal (17, 18).

#### 2.5.3 Insulin Resistance Index

IR status was defined based on the homeostasis model assessment (HOMA), calculated by multiplying the value of fasting plasma insulin (U/ml) and fasting plasma glucose (mmol/L) and then dividing by 22.5 (22).

#### 2.5.4 Insulin Resistance Status

The pubertal transition from Tanner stage 1 to Tanner stage 3 or 4 is associated with IR (22). For prepubertal children, a score of HOMA-IR ≥2.6 (23) was classified as IR, while a score of less than 2.6 was classified as insulin sensitive. For pubertal children, a score of HOMA-IR ≥4.0 was categorized as IR, while a score of less than 4.0 was categorized as insulin sensitive (24).

#### 2.5.5 Metabolic Syndrome Definition

For children aged 10 to <16 years:

Metabolic syndrome was established based on the International Diabetes Federation (IDF) definition (25). It was considered present if the WC measurement was ≥90th percentile of the Malaysian children WC chart (26) with the presence of at least two of the following criteria: TG ≥1.7 mmol/L, HDL-C <1.03 mmol/L, systolic blood pressure (SBP) ≥130 mmHg and/or diastolic blood pressure (DBP) ≥85 mmHg, or FBG ≥5.6 mmol/L (25).

### 2.6 Statistical Analysis

The normality test for continuous data was determined using the Kolmogorov–Smirnov test. Means and standard deviations were calculated for continuous variables. In testing the normality assumption, four variables were found to have a high skewness: TG, TG : HDL-C ratio, FBG, and insulin; these variables were transformed with a natural log function. Comparison of means between two groups was conducted using independent-samples t-test, while for categorical variables, comparisons were made using chi-square test. Statistical significance was set at 0.05.

### 2.7 Exploratory Factor Analysis

The relationship between TG: HDL-C ratio and leptin:adiponectin ratio (LAR) with MetS components (WC, HOMA-IR, SBP, and DBP) was first examined by exploratory factor analysis (EFA). EFA gathered and divided highly correlated variables in the MetS diagnosis into a specific grouping. This grouping was then confirmed in the confirmatory factor analysis (CFA). Factor extraction was performed using principal component analysis subjected to varimax rotation. Factor extraction produces the minimum number of factors that retain the total variance in the original data as possible. The factor loading of a variable on a factor equals the Pearson correlation coefficient between that variable and the factor. Thus, higher factor loadings represent more correlation between the variable and the factor. Additionally, variables grouped on the same factor is strongly correlated. Hence, represent the factor extracted. For example, the grouping of obesity markers (WC, percentage body fat, BMI z-scores) may be interpreted as the obesity group. Only variables with a factor loading of at least 0.3 (sharing at least 10% of the variance with a factor) were used for interpretation (27). The eigenvalues give information about potential components/factors and their relative explanatory power. In this study, factor extracted is considered valid if the eigenvalues are ≥1.0 (28). The Kaiser–Meyer–Olkin statistic >0.5 was used as a measure of sampling adequacy, and the Bartlett test of sphericity <0.001 was used as a measure of the necessity to perform a factor analysis (29).

EFA was first performed using all traditional variables (WC, DBP, SBP, FBG, TG, and HDL-C) from IDF definition with HOMA-IR and fasting insulin (model 1) as indicators of IR group. Secondly, EFA was performed with all variables from model 1 with the addition of commonly used obesity markers (BMI z-scores and percentage body fat) (model 2) because a minimum of the two variables is later needed to construct each group in the CFA. Additionally, TG : HDL-C ratio and LAR were also included in this EFA. Since factor analysis extracts factors due to the interrelatedness of measured variables (30), in the third EFA (model 3), individual variables of TG : HDL-C ratio and HOMA-IR, that is, TG, HDL-C, fasting insulin, and FBG, were removed from model 2.

### 2.8 Confirmatory Factor Analysis

**Figure 2:**
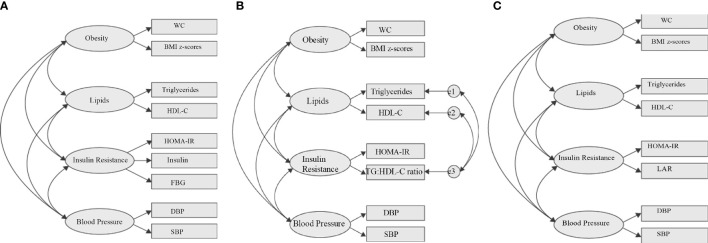
Hypothesized metabolic syndrome factor structure. **(A)** Four-factor correlated model based on the IDF definition. **(B)** Four-factor correlated model replacing fasting blood glucose with TG: HDL-C ratio to the IR component. **(C)** Referenced four-factor correlated model replacing fasting blood glucose with LAR to the IR component. WC, waist circumference; FBG, fasting blood glucose; DBP, diastolic blood pressure; SBP, systolic blood pressure; LAR, leptin:adiponectin ratio; e, residual covariance; HDL-C, high-density lipoproteincholesterol; HOMA-IR, homeostasis model assessment-estimated insulin resistance.

CFA was performed to confirm the IR group from EFA and to determine the best model to represent the MetS structures in children with obesity. We used SEM that utilized maximum likelihood estimation in Amos 21.0 (SPSS Inc., Chicago, IL) to develop our CFA models. SEM integrated CFA and path analysis (31). In this approach, MetS structure is visually constructed by correlating the four core risk factors (obesity, lipids, IR, and BP) with specific parameters or indicators measuring each risk factor (**Figure 2**). The analysis provides standardized factor loading that estimates the strength of the relationships between the core risk factors, between risk factors and parameters or indicators measuring each risk factor, and goodness-of-fit indices that indicate the adequacy of the model (32).

Firstly, we tested the basic MetS model from IDF definition using all traditional variables (WC, DBP, SBP, FBG, TG, and HDL-C), with HOMA-IR and fasting insulin (**Figure 2A**) as indicators of the IR group. Secondly, using the same basic IDF MetS model, we replace FBG with TG : HDL-C ratio as IR indicator (**Figure 2B**). Thirdly, using the same basic IDF MetS model, we replace FBG with LAR as IR indicator (**Figure 2C**). This third model is the reference model. The fit of individual model was determined using i) root mean square error of approximation (RMSEA) (threshold, <0.1) (33), ii) the comparative fit index (CFI) (threshold, >0.90) (33), and iii) Tucker–Lewis index (TLI) (threshold, >0.90) (34). Two-sided P values <0.05 were considered to be significant. We compared the goodness of fit of the first two models with the reference model (LAR) using i) goodness of fit (GFI) (threshold, > 0.9) (33), ii) Akaike’s information criterion (AIC) (35), iii) Bayesian information criterion (BIC) (35), and iv) Expected Cross-Validation Index (ECVI) (36). The model having smaller AIC, BIC, and ECVI values and closer to the LAR model is considered the preferred and parsimonious (a simple model with great predictive power) model.

All statistical analyses were run using the IBM Corp. Released in 2013. IBM SPSS Statistics for Windows, Version 22.0. Armonk, NY: IBM Corp. and AMOS software (ver21.0, IBM Corp., Armonk, NY, USA).

## 3 Results

### 3.1 General Characteristics of the Children With Obesity

The general characteristics and anthropometric measures of children included in this study are presented in **Table 1**. This study included 524 children with obesity ranging between 10 and 16 years old. More girls have reached puberty (92.4%) compared to boys (56%). The majority of the children (77.7%) were Malay followed by 10.3% of Indian ethnicity, 7.6% of Chinese ethnicity, and 4.4% of other minority ethnicities. Boys were mostly obese (52%) by BMI z-scores compared to the girls. The majority of the children (≈60%) were found to be abdominally obese.

**Table 1:** Characteristic and anthropometric measures of children with obesity.

	Boys	Girls	X^2^	p-value	All
n (%)	249 (47.5)	275 (52.5)			524
Mean age	12.4 ± 1.9	12.8 ± 1.9		0.02^a^	12.6 ± 1.9
Pubertal status					
n (%)					
Pre-pubertal	110 (44)	21 (7.6)	92.8	<0.001^b^	131 (25)
Pubertal	139 (56)	254 (92.4)			393 (75)
Ethnicity, n (%)					
Malay	185 (74.3)	222 (80.7)			407 (77.7)
Chinese	24 (9.6)	16 (5.8)			40 (7.6)
Indian	30 (12)	24 (8.7)	4.74	0.19^b^	54 (10.3)
Others	10 (4)	13 (4.7)			23 (4.4)
Anthropometric measures					
Obesity status					
BMI z score >1	79 (31.7)	128 (46.5)	12.63	0.002^b^	207 (39.5)
SD					
BMI z score ≥2	129 (51.8)	117 (42.5)			246 (46.9)
SD BMI z score ≥3	41 (16.5)	30 (10.9)			71 (13.5)
SD Abdominal obesity					
WC< 90th centile	97 (39)	96 (34.9)			193 (36.8)
			0.911	0.34^b^	
WC≥ 90th centile	152 (61.0)	179 (65.1)			331 (63.2)
BMI (mean ± SD)	26.7 ± 4.8	27.1 ± 4.9		0.4^b^	26.9 ± 4.9
BMI z score (mean ± SD)	2.36 ± 0.7	2.1 ± 0.7		<0.001^b^	2.2 ± 4.9
WC (cm) (mean ± SD)	83.9 ± 11.6	81 ± 10.6		0.003^b^	82.4 ± 4.9
Percentage body fat (%) (mean ± SD)	37.04 ± 7.2	40.6 ± 6.2		<0.001^b^	38.9 ± 6.9

WC, waist circumference; BMI, body mass index; SD, standard deviation.

a Independent-samples t-test.

b Pearson chi-square test.

Looking at the biochemical profile (**Table 2**), boys demonstrated higher FBG (4.87 ± 0.7 mmol/L vs. 4.79 ± 0.38 mmol/L) compared to the girls. Whereas girls were found to have higher leptin and LAR with 12.4 ± 8.6 mmol/L and 2.23 ± 1.7 mmol/L, respectively (both P < 0.001) compared to boys. With regard to clinical measures (**Table 3**), 6.9% (n = 36) of the children had MetS, about 40% of the children had IR, and ≈60% had AN.

**Table 2:** Biochemical measures.

Biochemical profile	Boys	Girls	p-value	All
(mean ± SD)				
Fasting blood glucose+ (mmol/L)	4.87 ± 0.7	4.79 ± 0.38	0.01	4.8 ± 0.37
Total cholesterol (mmol/L)	4.17 ± 0.67	4.17 ± 0.66	0.99	4.17 ± 0.67
Triglycerides (mmol/L)+	1.03 ± 0.56	0.95 ± 0.41	0.06	0.99 ± 0.49
HDL-C (mmol/L)	1.05 ± 0.21	1.05 ± 0.19	0.97	1.1 ± 0.2
HbA1c (%)	5.15 ± 0.3	5.15 ± 0.3	0.92	5.16 ± 0.3
Insulin (µU/ml)+	16.9 ± 11.4	17.9 ± 9.6	0.24	17.4 ± 10.5
Adiponectin (µg/ml)	6.25 ± 2.7	6.3 ± 2.6	0.8	6.27 ± 2.6
Leptin (ng/ml)	8.2 ± 5.9	12.4 ± 8.6	<0.001	10.39 ± 7.8
LAR	1.53 ± 1.33	2.23 ± 1.7	<0.001	1.89 ± 1.6
TG: HDL-C ratio+	1.04 ± 0.65	0.9 ± 0.46	0.05	0.99 ± 0.56

SD, standard deviation; LAR, leptin:adiponectin ratio.

+ Parameters not normally distributed were log transformed for statistical analysis; however, the actual untransformed values are reported.

**Table 3:** Clinical measures.

	Boys	Girls	X^2^	P value	All
SBP (mmHg) (mean ± SD)	107.7 ± 13	106.6 ± 12.2		0.342	107 ± 12.8
DBP (mmHg) (mean ± SD)	66.61 ± 10.57	67.28 ± 10.7		0.478	66.9 ± 10.6
Metabolic syndrome	16 (6.4)	20 (7.3)	0.147	0.702	36 (6.9)
[n (%)]					
Non-metabolic	233 (93.6)	255 (92.7)			
Syndrome [n (%)]					
Insulin resistance	100 (40.2)	107 (38.9)	0.09	0.77	207 (39.5)
[n (%)]					
Insulin sensitive [n (%)]	149 (59.8)	168 (61.1)			317 (60.5)
Presence of acanthosis nigricans [n (%)]	155 (62.2)	153 (55.6)	2.36	0.13	308 (58.9)
Absence of acanthosis nigricans [n (%)]	94 (37.8)	122 (44.4)			216 (41.1)

SBP, systolic blood pressure; DBP, diastolic blood pressure; SD, standard deviation.

### 3.2 Clustering of TG: HDL-C Ratio and Leptin: Adiponectin Ratio With Insulin Resistance Markers

**Table 4** displays the grouping of variables by EFA. Each model extracted three or four factors or groups with acceptable total variance of >60%, supporting the multifactorial component of MetS. EFA was performed in this study primarily to see the grouping of TG : HDL-C ratio with IR markers that will indicate the correlation of TG : HDL-C ratio with the IR group in the MetS component. Looking at the IR group in model 1, the IR group (factor 3) is presented by the grouping of HOMA-IR, FBG, and HDL-C with factor loadings of ≥0.3 that may indicate the correlation of lipids with the IR group. Interestingly, in model 2 (addition of percentage body fat, BMI z-scores, TG : HDL-C ratio, and LAR), lipid markers (TG, HDL-C, and TG : HDL-C ratio) were grouped with IR markers (HOMA-IR and fasting insulin) but not FBG (factor 2). Additionally, as expected, the classical IR markers (HOMA-IR, fasting insulin, and FBG) group together (factor 4), as both fasting insulin and FBG were directly correlated with HOMA-IR. Whereas LAR grouped with obesity (WC, PBF, BMI z-scores) and IR markers (HOMA-IR and fasting insulin) rather than only IR markers. This is probably because i) LARs are adipokines and directly related to adipose tissue and ii) previous bivariate correlation analysis has shown that LAR was highly correlated with WC. In the third EFA (model 3), individual variables of TG : HDL-C ratio and HOMA-IR, that is, TG, HDL-C, fasting insulin, and FBG, were removed. Profoundly, TG : HDL-C ratio and HOMA-IR were distinctly grouped in factor 3 with a strong standardized factor loading that may indicate the IR group. The factor loading for TG : HDL-C ratio and HOMA-IR was 0.904 and 0.664, respectively. Similar to model 2, LAR consistently grouped with the HOMA-IR and obesity markers (WC, PBF, BMI z-scores).

**Table 4:** Factor loadings of traditional variables with or without TG: HDL-C ratio, LAR, PBF, BMI z-scores, and HOMA-IR.

	Model 1 (IDF + HOMA-IR+ Insulin)			Model 2 (Adding PBF, BMI z scores, TG: HDL-C ratio, and LAR)				Model 3 (Removal of TG, HDL-C, Insulin, and Glucose)		
	Factor 1	Factor 2	Factor 3	Factor 1	Factor 2	Factor 3	Factor 4	Factor 1	Factor 2	Factor 3
	Obesity/IR	BP	IR	Obesity/IR	Lipids/IR	BP	IR	Obesity/IR	BP	IR
Triglycerides^+^	0.609				0.891					
HDL-C	0.472		-0.507		0.51					
HOMA-IR	0.863		0.304	0.464	0.306		0.73	0.407		0.664
Fasting insulin^+^	0.863			0.519	0.327		0.655			
Fasting blood glucose			0.823				0.771			
TG : HDL-C ratio					0.969					0.904
Leptin:adiponectin				0.609				0.591		
Waist circumference	0.478	0.611		0.773		0.385		0.773	0.381	
Percentage body fat				0.848				0.877		
BMI z scores				0.819				0.832		
Systolic blood pressure		0.905				0.886			0.906	
Diastolic blood pressure		0.894				0.906			0.914	
Variance explained (%)	29.43	26.48	13.83	24.64	18.83	15.3	13.9	32.76	23.51	17.37
Cumulative variance	29.43	55.92	69.75	24.64	43.46	58.77	72.74	32.76	56.27	73.64

BP, blood pressure; IR, insulin resistance; LAR, leptin:adiponectin ratio; PBF, percentage body fat; TG, triglyceride; IDF, International Diabetes Federation; HOMA-IR, homeostasis model assessment-estimated insulin resistance; HDL-C, high-density lipoprotein cholesterol.

^+^Skewed distributions were logarithmically transformed. Only factor loadings ≥0.3 are shown in the table to improve clarity.

### 3.3 Evaluation of TG: HDL-C Ratio as Insulin Resistance Component of Metabolic Syndrome

**Figure 3:**
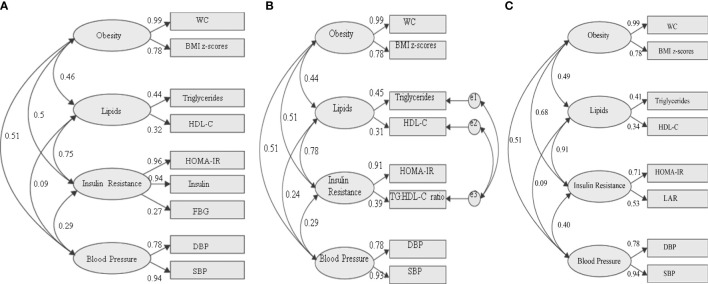
Three different models of metabolic syndrome structures with changed variables in the IR group. **(A)** Fasting blood glucose as measures of IR [fitness indices: P value (<0.05) = <0.001; RMSEA (<0.10) = 0.095; CFI (>0.9) = 0.957; TLI (>0.9) = 0.926]. **(B)** TG: HDL-C ratio as a measure of IR [fitness indices: P value (<0.05) = <0.001; RMSEA (<0.10) = 0.071; CFI (>0.9) = 0.987; TLI (>0.9) = 0.969]. **(C)** LAR as a measure of IR fitness indices: P value (<0.05) = <0.001; RMSEA (<0.10) = 0.064; CFI (>0.9) = 0.978; TLI (>0.9) = 0.955. The standardized factor loadings shown for all models are all statistically significant (P < 0.001). WC, waist circumference; FBG, fasting blood glucose; DBP, diastolic blood pressure; SBP, systolic blood pressure; LAR, leptin:adiponectin ratio; e, residual covariance.

**Figure 3** illustrates 2 competing models and 1 reference model of MetS with change variable to the IR risk factor. The first model used traditional variables from the IDF definition with HOMA-IR and fasting insulin (**Figure 3A**). This model was statistically significant, and all the goodness of fit indices have achieved the threshold value with CFI = 0.957, TLI = 0.926, and RMSEA = 0.095. Therefore, this confirms that the proposed construct of MetS is valid. Then, the correlation between the core factors/group (obesity, lipids, IR, and BP) can be scrutinized. Subsequently, the correlation between individual variables and the specific group can also be evaluated. Looking at the correlation between groups, the IR group was strongly correlated with the lipid group (standardized factor loading = 0.75) and moderately correlated with the obesity group (standardized factor loading = 0.46). Additionally, the obesity group also had a moderate correlation with the BP group (standardized loading = 0.51). The BP factor appeared to have weak correlations with the IR group (standardized factor loading = 0.29) and lipid group (standardized factor loading = 0.09). Looking at the IR group, FBG shows a weak standardized factor loading (0.27) indicating a weak correlation to the IR group.

We then tested a model in which FBG was replaced with TG : HDL-C ratio in the IR group. Since TG and HDL-C were directly correlated with TG : HDL-C ratio, we allow the residual errors to covary (**Figure 3B**). In CFA, a minimum of 2 variables is required for each group. However, in the first model, 3 variables (HOMA-IR, fasting insulin, and FBG) were required to achieve the desired goodness of fit. In contrast, in the second model, HOMA-IR and TG : HDL-C ratio were sufficient to construct the modeling. The model was statistically significant, and all the goodness of fit indices achieved the threshold value with higher CFI and TLI compared to the IDF model. The values of CFI and TLI for this model are 0.969 and 0.071, respectively. Additionally, this model has lower RMSEA (0.071) compared to that of the IDF model, indicating that this may be a better MetS model than the current IDF definition. Similar to the IDF model, the IR group was strongly correlated with the lipid group (standardized factor loading = 0.78) and moderately with the obesity group (standardized factor loading = 0.50). The obesity group also had a moderate correlation with the BP group (standardized factor loading = 0.51). The BP group appeared to have weak correlations with the IR (standardized factor loading = 0.29) and lipid (standardized factor loading = 0.24) group. Looking at the IR group, TG: HDL-C ratio shows a better-standardized factor loading (0.39) compared to FBG (0.27) in the IDF model.

Then, we tested our reference model in which LAR was one of the IR measures (**Figure 3C**). The model was statistically significant, and all the goodness of fit indices have achieved the threshold values, with CFI = 0.978, TLI = 0.955, and RMSEA = 0.064. Similar to the previous models, the IR group was strongly correlated with the lipid group (standardized factor loading = 0.91) and moderately with the obesity factor (standardized factor loading = 0.68). The obesity group also had a moderate correlation with the BP group (standardized loading = 0.51) and IR group (standardized factor loading = 0.4). The BP group appeared to have weak correlations with lipid factor (standardized factor loading = 0.09). Looking at the IR group, LAR shows a higher standardized factor loading (0.53) than FBG and TG: HDL-C ratio.

Finally, we compare the model fit indices of the two competing MetS model with the reference model (**Table 5**). The fit indices will determine the parsimonious model (a simple model with great predictive power). The TG: HDL-C ratio model shows a comparable GFI value to the reference model (LAR) compared to the FBG model. The GFI value was 0.98, 0.979, and 0.952, respectively. Similarly, the TG: HDL-C ratio model shows comparable AIC, BIC, and consistent AIC (CAIC) values to the reference model. Whereas the MetS model with FBG shows about 2-fold higher AIC, BIC, and CAIC values than the model with LAR, indicating the least fit model of the proposed MetS structures. Likewise, ECVI and the 90% ECVI also demonstrate an equal observation with other fit indices.

**Table 5:** Comparison of model fit indices for the proposed models.

Model	GFI	AIC	BIC	CAIC	ECVI	90% CI ECVI
Model 1 (FBG)	0.952	167.72	269.99	293.996	0.321	0.262, 0.394
Model 2 (TG: HDL-C ratio)	0.980	91.88	194.15	218.15	0.176	0.144, 0.222
Model 3 (LAR)	0.970	88.4	182.15	204.16	0.169	0.138, 0.215

FBG, fasting blood glucose; LAR, leptin:adiponectin ratio; GFI, goodness of fit; AIC, Akaike’s inclusion criterion; BIC, Bayesian information criterion; CAIC, consistent AIC; ECVI, Expected Cross-Validation Index.

Parsimonious model is indicated by relatively higher GFI and relatively smaller AIC, CAIC, and ECVI. Model 2 shows comparable fitness with the reference model (model 3).

## 4 Discussion

We used SEM to examine the usefulness of TG: HDL-C ratio as an IR surrogate in the diagnosis of MetS in children with obesity. Firstly, we have shown by EFA that TG: HDL-C ratio distinctively grouped with HOMA-IR, indicating a strong correlation between TG : HDL-C ratio and HOMA-IR, hence representing the IR group. This finding is not surprising, as numerous studies have shown the correlation between TG : HDL-C ratio and HOMA-IR using Pearson correlation or standard regression analysis (37, 38). However, standard Pearson or regression analysis is a measure of the association between two variables that indicates that the value of one variable changes reliably in response to changes in the value of the other variable. Whereas factor analysis assumes that the relationship (correlation) between variables is due to a set of underlying factors (latent variables) that are being measured by the variables. For example, in this study, the grouping of TG: HDL-C ratio with HOMA-IR may indicate that TG: HDL-C ratio is a measure of IR.

Secondly, CFA was performed to confirm the grouping of TG: HDL-C ratio and HOMA-IR from EFA and to compare whether FBG or TG: HDL-C ratio is a better measure of IR in the diagnosis of MetS in children with obesity. The established and widely used IR surrogate, the HOMA-IR calculations, requires the measurement of fasting insulin and FBG. Due to insulin instability, blood that is collected for insulin measurement must be kept cold and processed immediately, and the plasma is frozen as soon as the blood is withdrawn. Furthermore, measuring insulin is costly (39), and it is not a routine test (40). Therefore, there is a need to search for a simple, reliable, and applicable surrogate marker to measure IR especially among children with obesity. LAR is considered a stable and creditable obesity-IR marker in the pediatric population. The utility of LAR as an IR surrogate in children has been investigated in several studies (41, 42). Adipokines have been recommended as adipose tissue biomarkers by IDF in their “platinum standard” definition of MetS for research (25). Adipokines’ inclusion may better reflect adipose tissue function, since abdominal obesity is obligatory for IDF pediatric MetS diagnosis. However, leptin and adiponectin tests are expensive and not routinely tested. Therefore, our model that consists of LAR as an IR surrogate was considered as a reference model.

Our CFA model shows that MetS structure with TG : HDL-C ratio exhibits a better model fit than FBG and is closer to LAR. TG : HDL-C ratio shows higher standardized factor loading to the IR group than FBG, indicating that TG : HDL-C ratio is more correlated to the IR group than FBG (the standardized factor loading was 0.27 and 0.39, respectively). In contrast, when tested on adult males (≥40 years old), Shen et al. (43) demonstrated acceptable standardized factor loading (≥0.3) of FBG and post-challenge glucose in which the IR group was presented by fasting insulin and post-challenge insulin. Although impaired fasting glucose (IFG) was shown to predict diabetes mellitus in adults (44, 45), a similar finding has not yet been proven among children. Hagman et al. (46) reported that at the current IFG cutoff (5.6–6.0 mmol/L) as proposed by the American Diabetes Association, children with obesity show similar acute insulin responsiveness to glucose, insulin sensitivity index, and disposition index to children with normal FBG, suggesting that IFG in children may not be clinically useful as in the adult obese populations. Additionally, another study also reported that impaired insulin sensitivity was not present among youth in the prediabetic range (47, 48), hence signifying that IFG among children with obesity may be a less important driver of morbidity and mortality than that in adults.

Additionally, when comparing two or more competing models with different variables, the one with the smallest AIC and BIC values is the preferred model (35), indicating a preferred and parsimonious model (a simple model with great predictive power). Additionally, the ECVI measures the likelihood that a model would cross-validate across similar samples from the population (36). Models having smaller ECVI values are considered to have greater potential for replication. In our study, replacing TG: HDL-C ratio as an IR surrogate shows comparable GFI, AIC, and BIC with LAR. Whereas the least model fit was seen when using FBG as an IR surrogate. Moreover, a strong association was seen between the IR and lipid components in all of our hypothesized models, supporting the use of lipids as IR surrogate markers. Therefore, TG : HDL-C ratio can be considered as an IR surrogate marker in MetS components among children with obesity.

In addition, we found that about 60% of our children with obesity were abdominally obese. Thus, in agreement with previous studies, we also found a high prevalence of IR and AN (49). The association between abdominal obesity with IR is common and has been published in numerous studies (50, 51). Despite the high prevalence of abdominal obesity, less than 10% of the children had MetS. In contrast, a higher prevalence was reported among children with obesity in previous studies using the same MetS definition (52, 53). Very recently, using IDF definition, Bitew et al. (54) reported a pooled prevalence of MetS in low- and middle-income countries of 24.1% (95% CI: 16.90, 31.29) among children and adolescents with obesity. Similar to our study, almost 70% of the study subjects had abdominal obesity. Lower MetS prevalence was reported in our study probably due to the binary nature of the MetS diagnosis. Hence, even when the diagnostic criteria are increased, or even borderline, but still below the reference values of MetS diagnosis, the children will not be considered as having MetS. Likely, an individual with measurements in the MetS components just below the threshold for all five components may be at higher risk than someone who just exceeds the cutoffs in three components but has low or normal levels for the other two (55).

Realizing this, the IDEFICS (Identification and prevention of dietary and lifestyle-induced health effects in children and infants) research study proposed a continuous score combining the MetS components for children below 10 years old (56), which were considered at risk of MetS using the pediatric IDF definition. MetS scoring has the advantage of giving about equal chances for each of the components to contribute to the overall prevalence of the MetS. Although IDEFICS has a large sample population, the cohort was specifically targeted to pre-adolescent children aged below 10 years old. In contrast, our study population included children above 10 years old who are physiologically different from the IDEFICS study. Nevertheless, the study of MetS in Malaysian children is relatively new, and cutoff validation and continuous MetS scoring are beyond the scope of this paper. However, we agree that there is a need for future studies to verify the cutoff for the risk factors used in the diagnosis of pediatric MetS, thus improving the prognosis of MetS diagnosis.

In our study, all MetS models were determined to be valid and fit the data well, thus proving appropriateness to examine the potential of the TG : HDL-C ratio as an IR surrogate in the structure of MetS. Of note, percentage body fat was excluded from our CFA model because adding percentage body fat does not provide a good fit for our children’s data with obesity. The lack of fit is probably due to the established association between abdominal obesity (visceral fat) and MetS, which is best measured by WC (57). In comparison, percentage body fat is a weight percentage that results from total body fat, which consists of both subcutaneous and visceral fat. Thus, it does not support our factor structure of MetS.

In this study, we have provided the conceptual framework of MetS with the use of the TG: HDL-C ratio as IR surrogate using SEM in children with obesity. Although TG: HDL-C ratio has been extensively studied as an IR surrogate, we must emphasize that our employed measurement technique is novel compared with the existing literature. Our approach provides greater validity to the conclusion of TG : HDL-C ratio as an IR surrogate beyond standard correlation testing such as Pearson or standard regression analysis. Furthermore, we provide evidence on using TG: HDL-C ratio as an IR measure in the diagnosis of pediatric MetS. Additionally, due to the homogeneity of our study population (obesity), we have referred to the conceptual framework of MetS with the inclusion of LAR as obesity IR surrogate marker in addition to HOMA-IR, the general/universal IR surrogate marker. Furthermore, data from only children 10 years old and above were used in this study in parallel with the IDF definition for the diagnosis of pediatric MetS.

One of the limitations of this study was the cutoff value for HOMA-IR. Currently, there is no consensus on the HOMA-IR cutoff value. The common cutoff being used was between 1.14 and 5.56 (58, 59). This wide range may cause a variation in IR prevalence. Thus, it will affect the generalizability of the population. However, this was not the case in this study, as IR status was according to pubertal status, considering the influence of pubertal transition to IR. This study has validated the potential use of the TG: HDL-C ratio as an IR surrogate marker in the diagnosis of pediatric MetS, which may elevate future research of TG: HDL-C ratio for clinical application such as the validation of our proposed MetS definition across ethnicity. Importantly, future studies should apply the cutoff point of the TG : HDL-C ratio to the proposed pediatric MetS modeling, which may then be applied clinically.

In conclusion, IR is more prevalent than MetS in our study population of children with obesity. Thus, it is essential to assess the usefulness of the TG: HDL-C ratio as an IR component in the pediatric MetS structure. We confirmed that the TG: HDL-C ratio may replace FBG as IR surrogate marker in the MetS structure of children with obesity. We proposed targeted intervention for the individual at higher risk for future cardiometabolic risk in a situation where the resources are limited. This target group may be selected by utilizing our proposed MetS structures using TG: HDL-C ratio.

## Data Availability Statement

The raw data supporting the conclusions of this article will be made available by the authors without undue reservation.

## Ethics Statement

The studies involving human participants were reviewed and approved by the Medical Research and Ethics Committee (MREC), Ministry of Health Malaysia (NMRR-18-2749-41841). Written informed consent to participate in this study was provided by the participants’ legal guardian/next of kin.

## Author Contributions

AHM was the principal researcher and was responsible for the overall conception and design of project, coordinating with Ministry of Education and schools for data collection. AKNZI and RMWMZ were responsible for the logistics, collection, biochemical analysis of samples and computerization of all data. MYJ, FMZ, and JYHH were responsible for conceptualizing the clinical data collection and conducted the clinical examinations on the study subjects. FM was responsible for the logistics and collection and directed and oversaw the biological laboratory analysis. AY was responsible for sample size calculation and statistical analysis. All authors contributed to the article and approved the submitted version.

## Funding

This study was fully supported by a grant received from the Ministry of Health Malaysia (NMRR-18-2749-41841).

## Conflict of Interest

The authors declare that the research was conducted in the absence of any commercial or financial relationships that could be construed as a potential conflict of interest.

## Publisher’s Note

All claims expressed in this article are solely those of the authors and do not necessarily represent those of their affiliated organizations, or those of the publisher, the editors and the reviewers. Any product that may be evaluated in this article, or claim that may be made by its manufacturer, is not guaranteed or endorsed by the publisher.
